# A Preliminary Report on the Use of the Design of Experiments for the Production of a Synbiotic Yogurt Supplemented With Gluten Friendly^TM^ Flour and *Bifidobacterium infantis*

**DOI:** 10.3389/fmicb.2019.00226

**Published:** 2019-02-13

**Authors:** Antonio Bevilacqua, Barbara Speranza, Daniela Campaniello, Milena Sinigaglia, Maria Rosaria Corbo, Carmela Lamacchia

**Affiliations:** Department of the Science of Agriculture, Food and Environment, University of Foggia, Foggia, Italy

**Keywords:** synbiotic, gluten friendly, *Bifidobacterium* spp., centroid, desirability, multifactorial analysis of variance

## Abstract

The main goal of this paper was to design a synbiotic yogurt containing *Bifidobacterium infantis* and Gluten Friendly Flour^TM^; the proposed approach relies upon milk fermentation through the classical starter of yogurt (*Lactobacillus delbrueckii* subsp. *bulgaricus* and *Streptococcus thermophilus*) to avoid a strong production of acetic acid by bifidobacterial and inoculum of *B. infantis* after the fermentation. The research was divided in 3 steps. The aim of the first step was the optimization of fermentation kinetic by *L. delbureckii* and *S. thermophilus*, by combining the amount of flour (either Gluten Friendly Flour-GF- or Control Flour-CF) in milk, temperature and inoculum level; the factors were combined through a mixture design. As a result of this step, the best combination was pointed out: flour at 2.5 g/l; *L. delbrueckii* subsp. *bulgaricus* at 6 log cfu/ml; temperature at 37–40°C. The goal of the second step was to study the effect of flour (2.5 g/l) on the viability of *B. infantis*. GF prolonged the viability of the probiotic for 14 days. In the last step, a synbiotic yogurt, supplemented with GF and fermented with *L. delbureckii* and *S. thermophilus*, and then inoculated with *B. infantis*, was produced. The product was stored at 8 and 15°C. A positive effect of GF was found at 15°C, with *B. infantis* at 7.0 log cfu/g in GF sample and 5.5.5.7 log cfu/g in CF sample.

## Introduction

Nowadays consumers believe that foods can significantly contribute to their health and wellness (Mollet and Rowland, 2005; Hassani et al., 2016) and functional products meet this increasing demand of healthy diet, as they satisfy hunger and provide essential nutrients, but they can also prevent diseases and promote physical and mental wellness (Menrad, 2003).

Dairy products are functional foods since they are sources of calcium and they are vehicles of probiotic microorganisms (Plessas et al., 2012). Fermented milks are the classical carriers for probiotic microorganisms, due to the high consumers' preference for these beverages and for their proven health benefits (Batista et al., 2015). The survival of probiotics in fermented milks is a challenge, due to the influence of several intrinsic and extrinsic factors (Granato et al., 2010). There are several regulatory requirements for probiotic yogurts; the most important one is that the microorganisms are alive at the time of use, and the viable count must be at the level proven to confer a health benefit, i.e., at least 7 logcfu/g (Rosburg et al., 2010).

*Bifidobacterium* is a member of intestinal microbiota of mammals and is among the first microbial colonizers of the intestines of newborns; it plays key roles in the development of their physiology, including maturation of the immune system and use of dietary components. Some *Bifidobacterium* strains are probiotic microorganisms because of their beneficial effects (Hidalgo-Cantabrana et al., 2017).

Probiotic microorganisms can be added to fermented milks using different methods. They might be added as non-fermenting microorganisms after fermentation and cooling with the sole aim of being delivered through gastrointestinal tract (nonstarter probiotics), or be added as starter cultures to ferment milk base (starter probiotics) (Mohammadi et al., 2012).

Bifidobacteria were used to produce fermented milks, but they showed lower performances than lactobacilli, thus hindering their possible applications (Prasanna et al., 2012b). Moreover, their growth and viability are challenges, as they require longer fermentation times, strict anaerobic conditions and a low redox potential (Prasanna et al., 2012a). In addition, acetic acid and the lack of acetaldehyde could lead to off-flavors and off-odoursc(Mohammadi et al., 2012). The *Bifidus* fermentation pathway produces acetic acid and lactic acid at a ratio 3:2, and the production of acetic acid at high levels could be responsible of a “vinegary taint” (Mortazavian et al., 2011). Therefore, it is not advisable to use bifidobacteria as the only starter for fermented milk, while they should be combined with other lactic acid bacteria.

The most popular way is to add the probiotic microorganisms together with adjunct lactic starter cultures (mainly, traditional yogurt bacteria: *Lactobacillus delbrueckii* ssp. *bulgaricus* and *Streptococcus thermophilus*), since the adequate fermentation in milk rarely occurs by probiotics alone.

Lamacchia and coworkers (Lamacchia et al., 2013, 2015) designed a new and innovative method (Gluten Friendly^TM^)(PCT/IB2013/000797) for grain seeds to reduce the immunogenicity of gluten *in vitro*; this approach is based on the use of microwave on hydrated wheat kernels. Microwaves induce structural modifications to endosperm content, and as a result, the immunogenicity is significantly reduced (Lamacchia et al., 2016). An additional effect of this treatment is the functional effect of Gluten Friendly^TM^ on microorganisms, like the antimicrobial activity toward *Salmonella* sp., and the prolonged the viability of *Lactobacillus acidophilus* La5 in a model system (Bevilacqua et al., 2016) and in a fermented milk during a refrigerated storage (Speranza et al., 2018). Moreover, Gluten Friendly^TM^ flour or bread promoted a partial restoration of gut microbiota of coeliac subjects both in batch systems and in a gut model system (Bevilacqua et al., 2016; Costabile et al., 2017), as well as the increase of mucin production (Lamacchia et al., 2018).

The aim of this research was to design a yogurt, fermented by *L. delbrueckii* subsp. *bulgaricus* and *S. thermophilus*, and supplemented with Gluten Friendly Flour and *Bifidobacterium infantis*. The research was divided into three different steps: (i) evaluation of the effects of Gluten Friendly flour on the acidification kinetics of the starter strains; (ii) study of the effects of flour on the viability of *B. infantis*; (iii) validation at laboratory level and production of a yogurt, supplemented with both flour and *B. infantis*.

## Materials and Methods

### Microorganisms and Raw Materials

*Bifidobacterium infantis* Bb02 was purchased from Chr. Hansen (Hørsholm, Denmark); the strain was grown in MRS broth (Oxoid, Basingstoke, UK) supplemented with 0.5% cysteine (Sigma-Aldrich, Milan, Italy) at 37°C for 24 h under anaerobic conditions. *Lactobacillus delbrueckii* subsp. *bulgaricus* DSM 20081 was purchased from the German Collection of Microorganisms (DSMZ, Braunschweig, Germany) while *Streptococcus thermophilus* was purchased from Clerici-Sacco Group (Como, Italy). *L. bulgaricus* and *S. thermophilus* were grown in MRS broth (37°C for 24 h; anaerobiosis). Before each experiment the strains were centrifuged at 4,000 g for 10 min and suspended in sterile distilled water (9 log cfu/ml).

Gluten Friendly Flour (GF) (Lamacchia et al., 2015) was prepared as described by Speranza et al. (2018). Gluten Friendly^TM^ method is an innovative approach to treat wheat grains. Wheat grains was dampened until reaching 15–18% humidity, and then heated through microwaves (approximately 1 min between 1,000 and 750 watts); microwave heating was followed by a slow evaporation of the water. This combination (heating/evaporation) was repeated until reaching a final temperature of 80–90°C, and a moisture degree of 13–13.5%. After the treatment, the kernels were cooled and dried at 24°C for 12–24 h. A flour not treated with microwaves was used as control (CF).

Fresh whole pasteurized homogenized cow's milk (3.35 g/l protein; 5.0 g/l carbohydrates; 3.75 g/l fats) was used. Before each experiment, lactobacilli, lactococci and spoiling microorganisms (enterobacteria, spore formers, and pseudomonads) were checked if they were below the detection limit (standard plate count).

### Evaluation of Acidification Kinetics of *L. delbrueckii* subsp. *bulgaricus* and *S. thermophilus*

*L. bulgaricus* and *S. thermophilus* were inoculated to 4, 6, and 8 log cfu/ml, depending on the experimental plan, in 15 ml pasteurized milk supplemented with either GF or CF (0.0–2.5–5.0 g/l); the samples were incubated at 30–45°C for 72 h and the pH was measured after 4, 6, 15, 18, 21, 24, 28, 30, 39, 48, and 72 h. Flour, inoculum and temperature were combined through a simplex centroid. The combinations of the design are in Table 1.

**Table 1:** Combinations of the centroid.

	**Coded values**			**Effective values**		
**Samples**	**Inoculum**	**Flour**	**Temperature**	**Inoculum (log cfu/ml)**	**Flour (g)**	**Temperature (°C)**
A	1	0	0	8	0	30
B	0	1	0	4	5	30
C	0	0	1	4	0	45
D	0.5	0.5	0	6	2.5	30
E	0.5	0	0.5	6	0	37.5
F	0	0.5	0.5	4	2.5	37.5

Four different designs were run: (a) *L. bulgaricus*+GF; (b) *L. bulgaricus*+CF; (c) *S. thermophilus*+GF; (d) *S. thermophilus*+CF.

The experiments were performed on two different samples; for each sample the measurements were repeated twice. The results were modeled as acidification (ΔpH) through the lag-exponential model (Van Gerwen and Zwietering, 1998; Baty and Delignette-Muller, 2004; Delignette-Muller et al., 2006), modified by Speranza et al. (2018):

$$\Delta \text{pH}=\{\begin{array}{ll} 0 & \text{t}\leq \alpha \\ \Delta {\text{pH}}_{\text{max}}-\text{log}\{1+\left(10^{\Delta \text{pH}}{\;}_{\max}-1\right) & \\ \;{\;}^{*}\exp[-d_{\text{max}}(\text{t-}\alpha )]\} & \text{t}>\alpha \end{array}$$

Or

ΔpH = Δ*pH*_max_ – log{1+ (10^Δ*pHmax*^ −1) ^*^ exp (–*d*_max_ *t*)}

where:

α is the time before the beginning of the acidification kinetic (h); d_max_ is the maximal acidification rate (1/h); ΔpH_max_ is the maximum level of acidification.

Then, ΔpH_max_ and α were analyzed through the theory of the Design of the Experiments, to assess the significant effect of temperature, inoculum and flour as well as of their interactions. The analysis was done through the software Statistica for Windows (Statsoft, Ulsa, Okhla). The significance of the whole approach, as well as of each variable, was evaluated through the adjusted regression coefficients, the mean square residual, and the Fisher test (*P* < 0.05). The effect of each factor of the design (inoculum, temperature, flour) was also evaluated through the individual desirability functions, estimated as follows:

$$d=\{\begin{array}{l} 0,\text{ y}\leq {\text{y}}_{\min}\\ \left(\text{y}-{\text{y}}_{\min}\right)/\left({\text{y}}_{\max}-{\text{y}}_{\min}\right)\;{\text{y}}_{\min}<\text{y}<{\text{y}}_{\max}\\ 1,\text{ y}\geq {\text{y}}_{\max} \end{array}$$

Where y_min_ and y_max_ are the minimum and maximum values of the dependent variable, respectively.

The desirability is a dimensionless parameter ranging from 0 to 1; it i the answer to the question: how much desired is a result? Generally, desirability is set to 0 for the lowest or the worst value of the dependent variable and 1 for the maximum or the most desired value (in this paper it was set to 0 for the lowest value of ΔpH_max_ and α and 1 for their maximal values). It is a mathematical function estimated by focusing on the effect of each factor *per* time, while the other variables/factors of the design were set to a constant value. For this research, the desirability profiles were built by setting the variables to the coded level 0.33 (inoculum to 5.3 log cfu/ml, temperature to 35°C, flour to 1.65 g/l).

The desirability profile has two main applications: (a) to study the effect of each variable, without the influence of possible interactive or synergistic effects; (b) to compare variables or parameters with different units.

### Effect of GF on the Viability of *Bifidobacterium infantis*

Saline solution was supplemented with either GF or CF (2.5 g/l) and inoculated with *B. infantis* to 7.70 log cfu/ml. The samples were stored at 4 and 37°C and analyzed after 1, 2, 4, 9, 14, and 21 days by pour plating (MRS Agar+cysteine, 37°C for 48–72 h under anaerobic conditions).

Saline solution without flour but inoculated with *B. infantis* was used as control. The experiments were performed twice on two independent samples.

### Yogurt

Three different productions of yogurt were prepared as follows: milk supplemented with Gluten Friendly flour (2.5 g/l) (GF), control flour (2.5 g/l) (CF) or without flour (CNT, control). Then, the three batches were inoculated with *L. delbrueckii* subsp. *bulgaricus* (6 log cfu/ml) and *S. thermophilus* (6 log cfu/ml) and incubated at 40°C and let to ferment until a pH 4.0 was attained.

After the fermentation, the batches were inoculated with *B. infantis* (6.5–6.8 log cfu/ml), divided in samples of 25–30 g in sterile 50 ml-tubes and stored at 8 and 15°C. Immediately after the inoculation of bifidobacteria and after 3, 7, 10, 14, and 17 days microbiological and chemico-physical analyses were done.

For microbiological analyses, the following media were used: MRS Agar and M17 Agar+lactose, incubated at 30°C or 44°C for 48–72 h for mesophilic and thermophilic lactobacilli and lactococci; MRSAgar+cysteine+NNLP antibiotic solution (**N**eomycin-**N**alidixic acid-**L**ithium chloride-**P**aromomycine: 2 g/l neomycin sulphate, 4 g/lparomomycine sulphate, 0.3 g/l nalidixic acid, and 60 g/l lithium chloride; all reagents were purchased from Sigma-Aldrich), incubated at 37°C for 48–72 h for bifidobacteria; Slanetz/Bartley Agar incubated at 37°C for 48 h, for enterococci; Plate Count Agar (PCA) incubated at 5 C for a week or 32°C for 48 h for psychrotrophic bacteria and mesophilic bacteria, respectively; Baird-Parker agar base, with egg yolk tellurite emulsion, incubated at 37°C for 48 h for staphylococci and *Micrococcaceae*; Pseudomonas Agar Base (PAB) with CFC Selective Supplement incubated at 25°C for 48 h for *Pseudomonas* spp.; Violet Red Bile Glucose Agar (VRBGA), incubated at 37°C for 24 h for *Enterobacteriaceae;* Violet Red Bile Agar (VRBA) incubated at 37°C or 42°C for 18–24 h for total and fecal coliforms, respectively; Sabouraud dextrose agar, supplemented with chloramphenicol (0.1 g/l) (C. Erba, Milan, Italy), incubated at 25°C for 48 h or 5 days, for yeasts and molds, respectively. All the media and the supplements were from Oxoid.

The viable count of *Bifidobacterium* was confirmed by a random isolation of some colonies, and microscopic examination.

pH was measured by a pH-meter and a_w_ through an AQUALAB CX-2 (Decagon Device, Pullman, WA, USA). Color was evaluated by a colorimeter Chroma Meter (Minolta, Japan) by measuring CIE L^*^ (lightness), a^*^ (redness), and b^*^ (yellowness) values.

The experiments were repeated twice on two independent samples. The results of *Bifidobacterium* were analyzed by a multifactorial analysis of variance and Tukey's test as the *post-hoc* comparison test. Storage time, temperature and kind of samples (control, GF or CF) were used as categorical predictors; the critical P-level was set to 0.05.

## Results

### Acidification Kinetics

**Figure 1:**
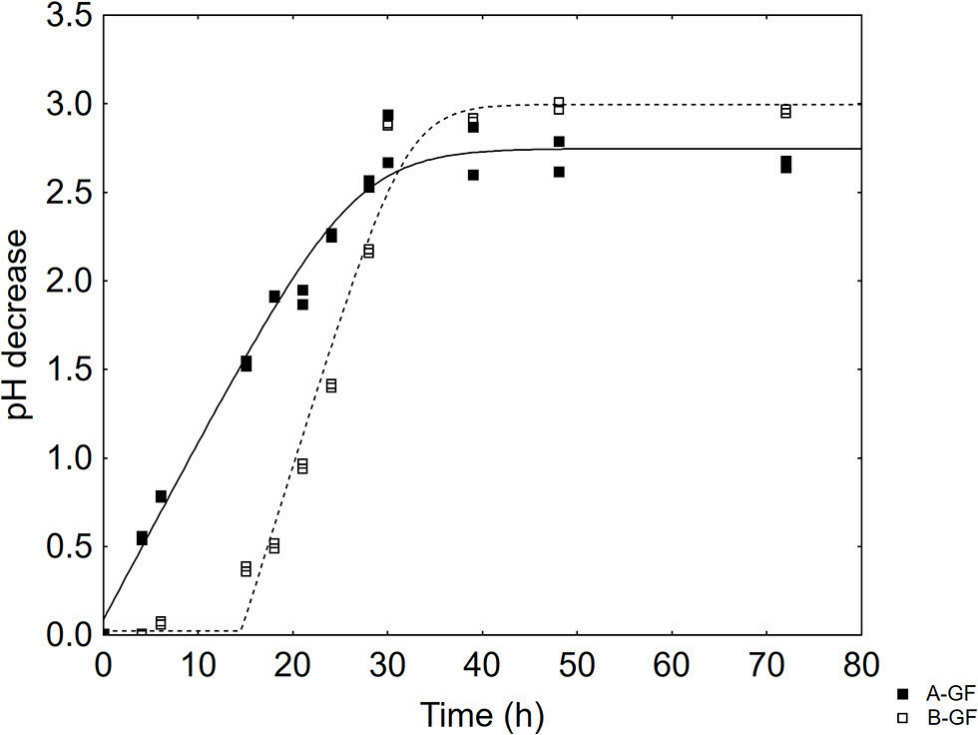
Acidification of *L. delbrueckii* subsp. *bulgaricus* in two selected combinations of the design. The lines represent the best fit through the lag-exponential equation. GF, Gluten Friendly Flour.

The aim of the first step was to study the effect of either GF or CF on acidification kinetics *L. delbrueckii* subsp. *bulgaricus* and *S. thermophilus*. The microorganisms experienced a sigmoid-like acidification (see as an example Figure 1), with an exponential-like step (fast decrease of pH) and a steady state (a maximum value of acidification); in some combinations, there was a preliminary step when pH was constant (a lag-like period).

**Figure 2:**
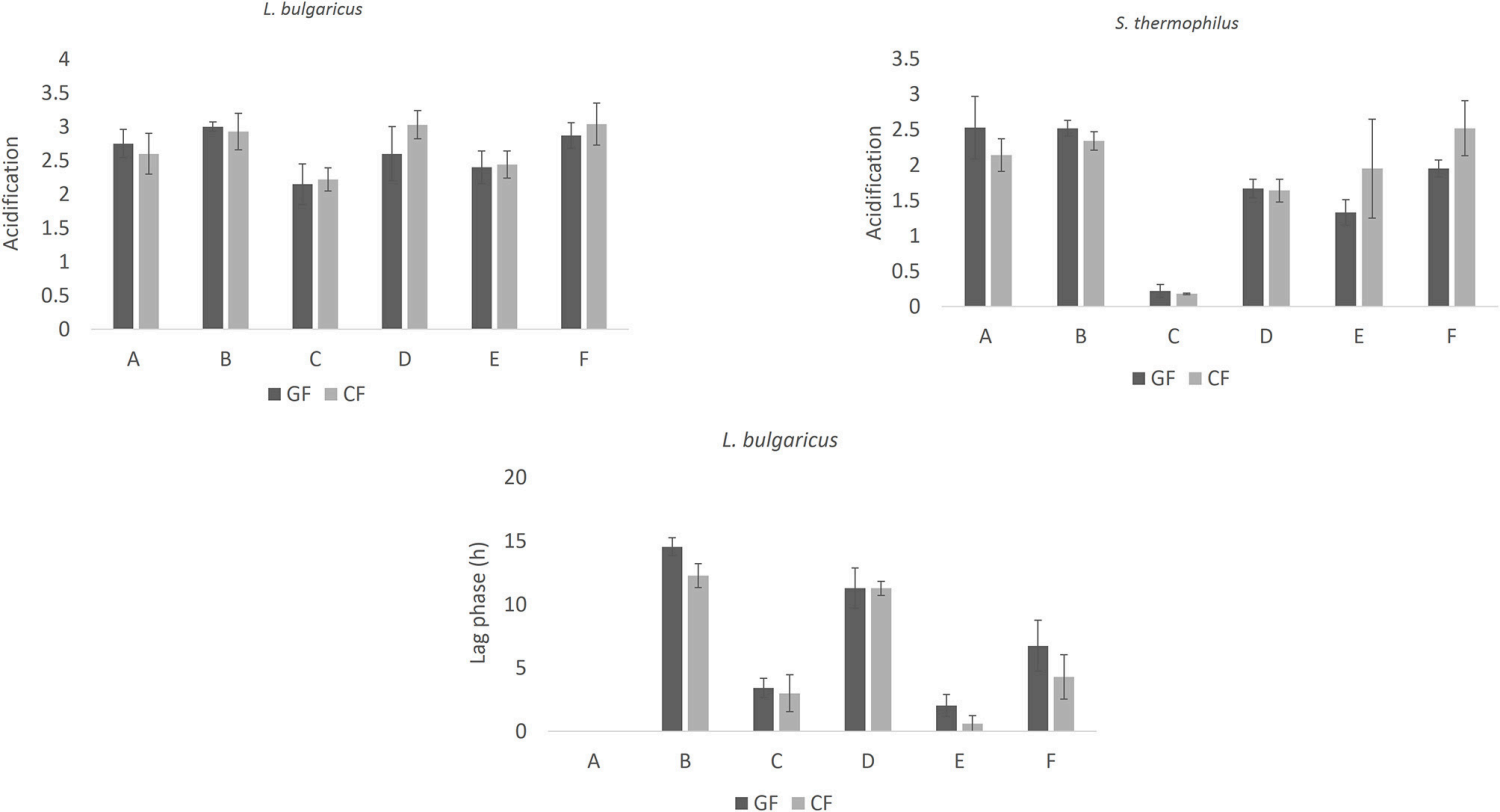
Parameters (mean values ± standard error) of the lag-exponential model for *L. delbrueckii* subsp. *bulgaricus* and *S. thermophilus* in the combinations of the design. Acidification: parameter ΔpH_max_; lag phase: parameter α. GF, Gluten Friendly Flour; CF, Control Flour.

Figure 2 shows the most important parameters of these kinetics: ΔpH_max_ (acidification), and α (time before the beginning of acidification kinetic) for *L. bulgaricus* and *S. thermophilus*. ΔpH_max_ was 2.5–3.0 for *L. bulgaricus* and 1.8–2.7 for *S. thermophilus*. This microorganism did not experience an acidification kinetic in the combination C. The parameter α was never found for *S. thermophilus*, while it was from 0 (combination A) to 14 h (combination B) for *L. bulgaricus*.

ΔpH_max_ and α were used as inputs to run a DoE analysis and to study the effect of flour, inoculum and temperature. The significance of these effects is in Table 2. The approach was highly significant for *L. bulgaricus*, as shown by the high regression coefficients for both GF and CF (from 0.947 to 0.961).

**Table 2:** Standardized effects of inoculum, flour and temperature on acidification (ΔpH_max_) of *L. delbrueckii* subsp. *bulgaricus* (Lb) and *S. thermophilus* (St) and on the time before the beginning of acidification (α) of *L. bulgaricus*. $R_{\text{ad}}^{2}$, adjusted regression coefficient.

	**ΔpH_**max**_Lb**	**α Lb**	**ΔpH_**max**_ St**
**GLUTEN FRIENDLY FLOUR**			
Inoculum	68.97	–^*^	12.43
Flour	75.24	20.88	12.39
Temperature	53.95	4.93	–
Inoculum by flour	−5.51	4.67	−3.44
Inoculum by temp.	–	–	–
Temp. by flour	6.14	−2.64	2.33
$R_{\text{ad}}^{2}$	0.947	0.950	0.835
**CONTROL FLOUR**			
Inoculum	70.09	–	6.81
Flour	78.88	19.98	7.45
Temperature	59.69	4.88	–
Inoculum by flour	5.83	6.82	–
Inoculum by temp.	–	–	–
Temp. by flour	10.39	−4.44	3.24
$R_{\text{ad}}^{2}$	0.961	0.956	0.645

* *Not significant*.

The statistical weight of the variables was the same for both GF and CF; acidification (ΔpH_max_) was affected by all factors as individual terms and by the interactions “inoculum/flour” and “temperature/flour,” but the most significant term was the individual effect of flour. The parameter α was affected by the individual terms of temperature and flour and by the interactive factors “inoculum/flour” and “temperature/flour.” On the other hand, the significance of the approach was lower for *S. thermophilus*.

**Figure 3:**
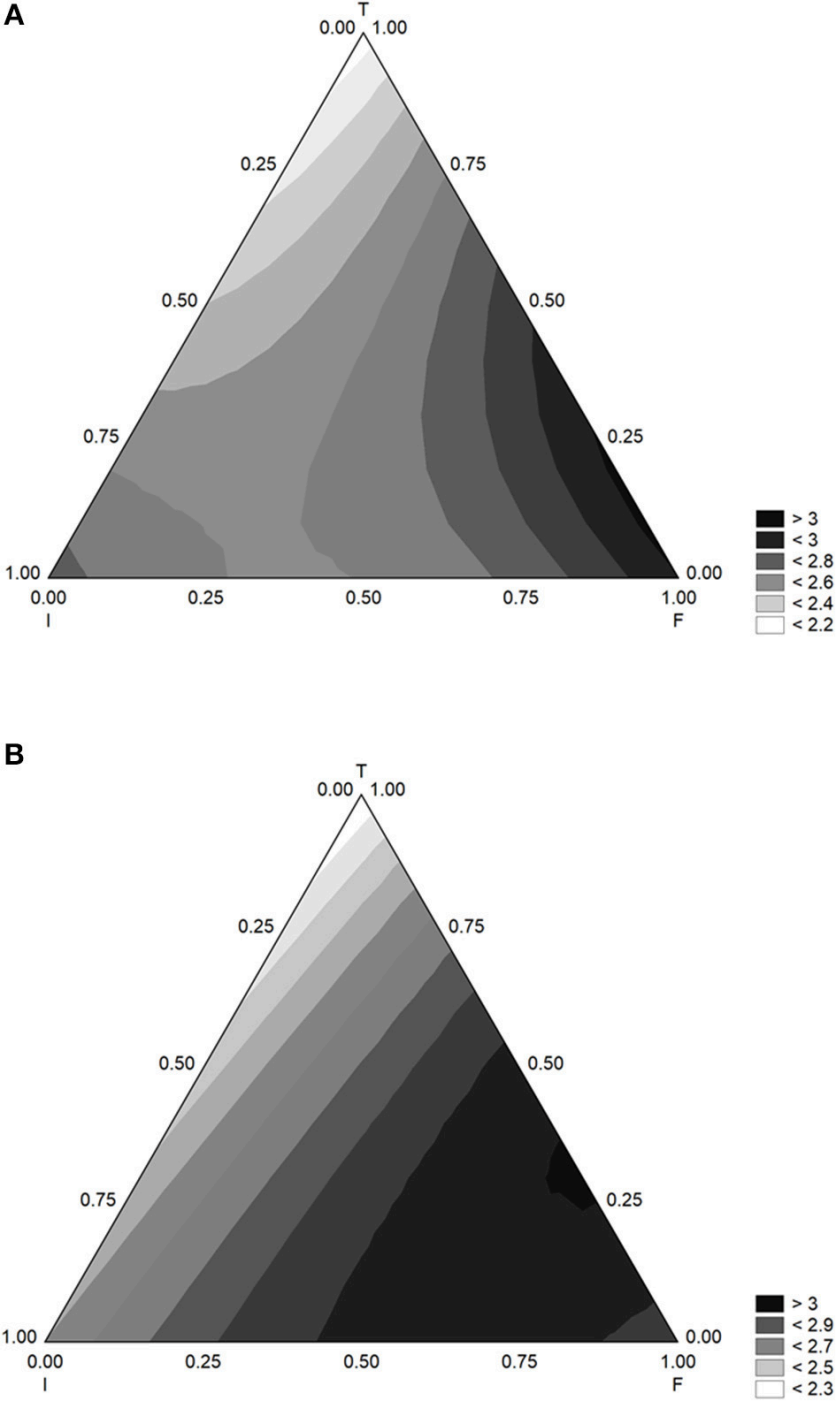
Triangular surfaces for the effect of flour (F), inoculum (I), and temperature (T) on the acidification (parameter ΔpH_max_) of *L. delbrueckii* subsp. *bulgaricus*. **(A)** Gluten Friendly Flour; **(B)** Control Flour.

A table of standardized effect offers a qualitative output; some details on the quantitative correlation of the factors with ΔpH_max_ and α can be achieved by mean of ternary plots. A ternary plot is a triangular-like graphs and shows the effects of three variables (inoculum, flour, and temperature) in a 2D space. Figure 3 shows the triangular plots for ΔpH_max_ with GF (A) and CF (B). The effects were similar, and the model predicted the maximum level of acidification (ΔpH 3) with flour at coded levels 0.5–1.0 (effective value from 2.5 to 5.0 g/l) and inoculum at 0.25–0.50 (effective values, 5–6 log cfu/ml); the effect of temperature was negative, as an increase caused a decrease of ΔpH_max_.

**Figure 4:**
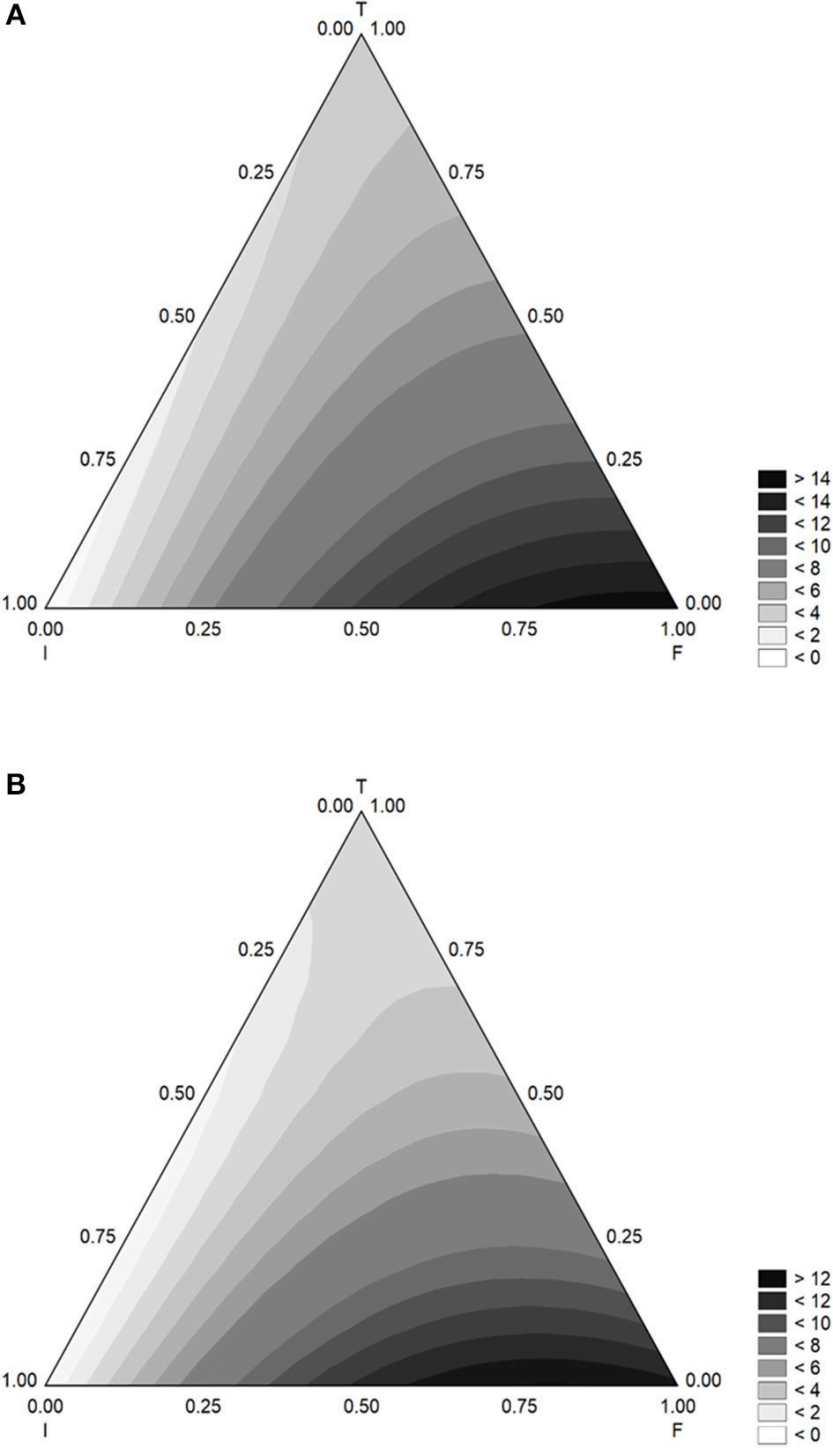
Triangular surfaces for the effect of flour (F), inoculum (I), and temperature (T) on the time before the beginning of acidification (parameter α) of *L. delbrueckii* subsp. *bulgaricus*. **(A)** Gluten Friendly Flour; **(B)** Control Flour.

The triangular plots for α are in Figure 4. The lag phase increased when flour increased and was maximum (12–14 h) for the coded level 1 (flour at 5.0 g/l); inoculum and temperature negatively acted, and the lag phase was at its relative minimum values (1 or 3 days) when inoculum or temperature were at the coded level 1.

**Figure 5:**
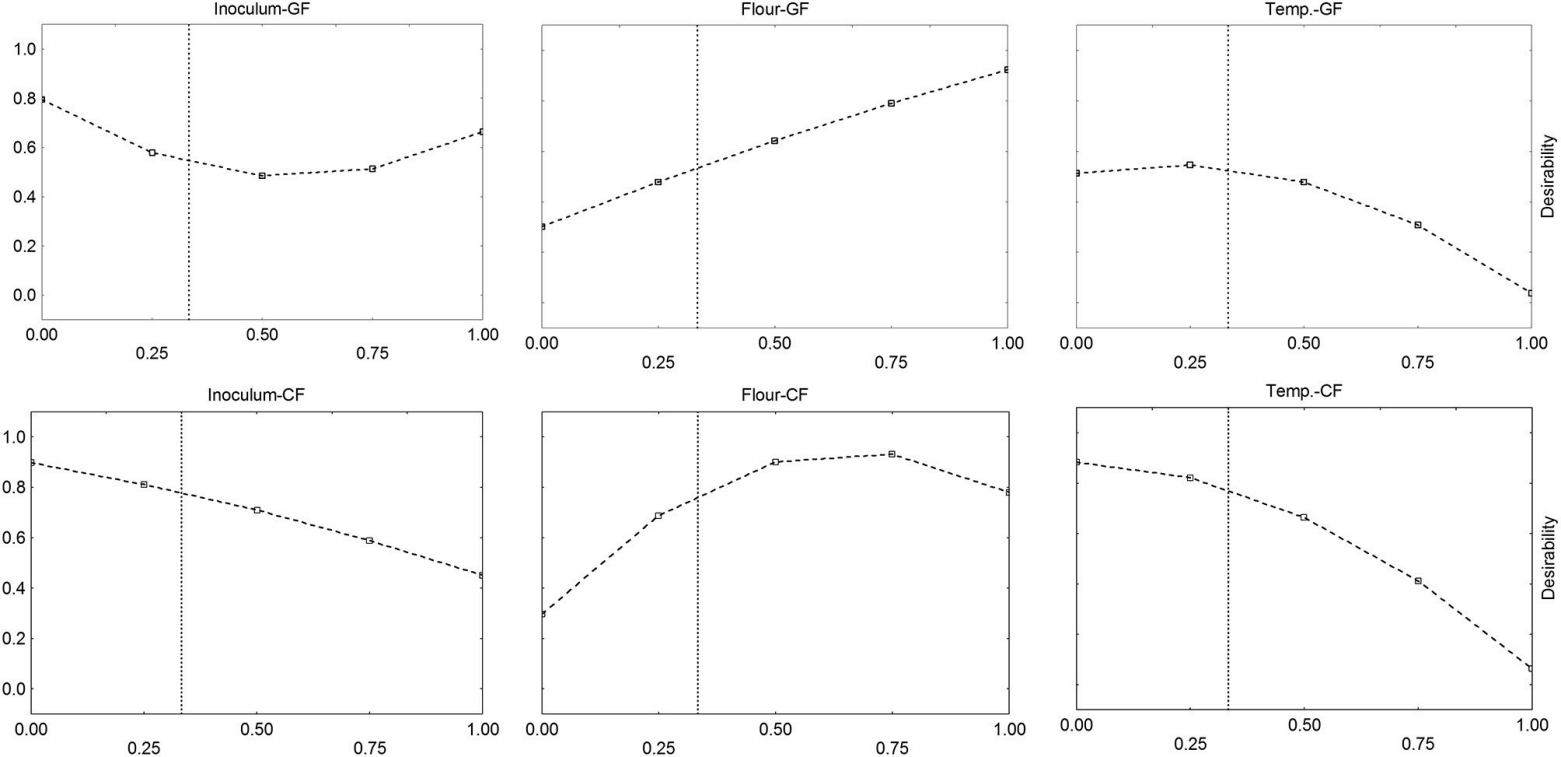
Desirability profiles for the effect of flour (F), inoculum (I), and temperature (T) on the acidification (parameter ΔpH_max_) of *L. delbrueckii* subsp. *bulgaricus*. GF, Gluten Friendly Flour; CF, Control Flour.

Ternary plots suffer a main drawback: they offer quantitative trends for interactions, but it is not possible to evaluate the effect of each factor alone. This goal can be achieved through the desirability profiles. The desirability profiles of the individual effects of inoculum, flour, and temperature on ΔpH_max_ are in Figure 5. The correlation inoculum/acidification was negative, as desirability decreased by increasing inoculum, while the correlation was positive for flour with slight differences between GF and CF. For GF the correlation flour/acidification was strictly linear, while for CF the correlation flour/acidification was quadratic, with a maximum for a coded level of flour at 0.75 (effective values 3.75 g/l). Finally, the effect of temperature was negative.

**Figure 6:**
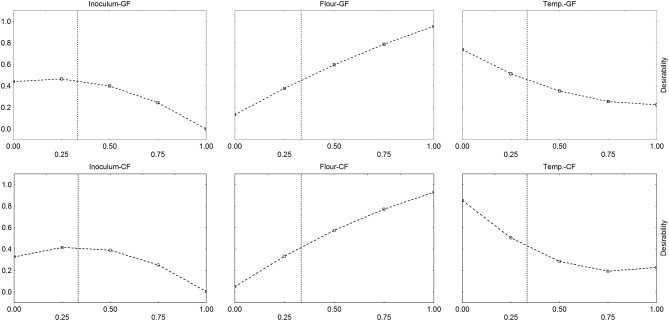
Desirability profiles for the effect of flour (F), inoculum (I), and temperature (T) on the time before the beginning of acidification (parameter α) of *L. delbrueckii* subsp. *bulgaricus*. GF, Gluten Friendly Flour; CF, Control Flour.

The same approach was used for the lag phase (Figure 6). Desirability profiles show that the desirability, i.e., the lag phase, increased by increasing both flour and temperature: the effect of flour was strong, as desirability increased from 0.1 to 1.0 by increasing flour to the coded level 0 (0 g/l) to 1 (5 g/l). On the other hand, the profiles for the temperature confirmed what found on the triangular plots: the correlation lag phase/temperature was negative, and the lag phase decreased by increasing temperature.

**Figure 7:**
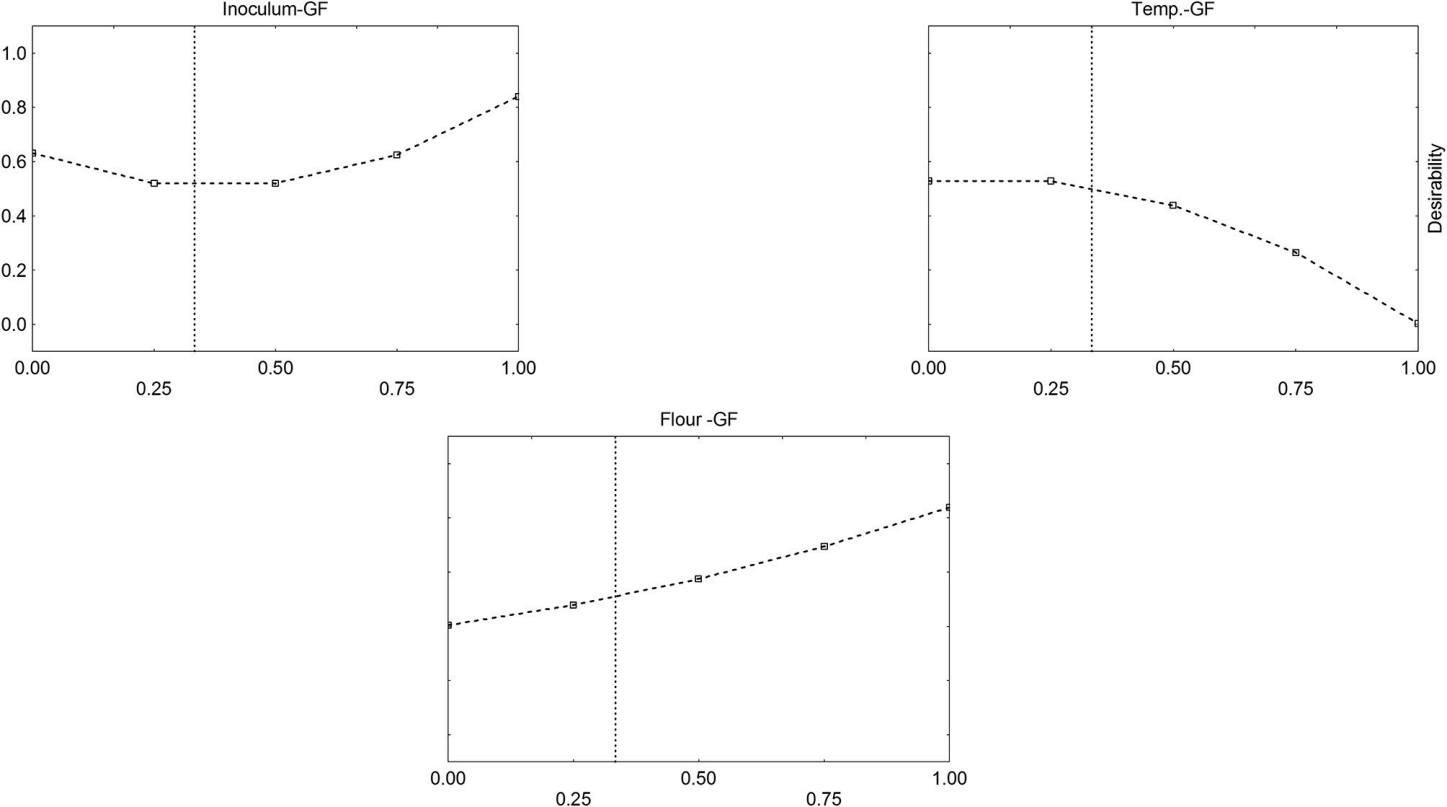
Desirability profiles for the effect of Gluten Friendly Flour (F), inoculum (I), and temperature (T) on the acidification (parameter ΔpH_max_) of *S. thermophilus*.

Desirability profiles and triangular plots were built for *S. thermophilus*, too (see as an example Figure 7); however, as reported above, the model was less significant, and the desirability profiles could not be used for an optimization process.

The last step of the statistical modeling was the optimization, i.e., the choice of the best combinations of the variables. For this research, the best combination would ideally result in the highest level of ΔpH_max_ and the lowest value of α (lag phase). However, the results of the DoE approach highlighted an uncoupling between the level of acidification and the lag phase: the combinations resulting in the highest level of acidification caused a significant prolongation of the lag phase. Therefore, a different goal was chosen: acidification of 2.0–2.5 with a lag phase of 6–7 h. The combination of all desirability profiles (saddle point approach) suggest that this goal could be achieved by setting the variables as follows:

- flour at 2.5 g/l
- *L. delbrueckii* subsp. *bulgaricus* at 6 log cfu/ml
- Temperature at 37–40°C

### Effect of GF on *B. infantis*

Before producing the synbiotic yogurt, the effect of flour was studied on *B. infantis*. The results are in Table 3. Both in the control and in the sample supplemented with CF, the microorganism was below the detection limit after 4 days, while a residual population was found in the sample with GF after 4 (5.08 log cfu/ml), 9 (4.08 log cfu/ml), and 14 days (3.60 log cfu/ml).

**Table 3:** Viable count of *B. infantis* (log cfu/ml) in saline solution (9 g/l NaCl) supplemented with either Gluten Friendly (GF) or Control Flour (CF) (2.5 g/l).

**Time (days)**	**CNT**	**CF**	**GF**
0	7.72 ± 0.23	7.72 ± 0.23	7.72 ± 0.23
1	5.41 ± 0.08	6.72 ± 0.13	6.15 ± 0.13
2	4.30 ± 0.17	5.54 ± 0.21	6.08 ± 0.21
4	–^*^	–	5.08 ± 0.19
9	–	–	4.08 ± 0.33
14	–	–	3.60 ± 0.21
21	–	–	–

*CNT, control (saline solution). Mean values ± standard deviation. The samples were incubated at 37°C*.

* *Below the detection limit (1 log cfu/ml)*.

### Yogurt

The synbiotic yogurt was produced through the classical starter strains and then supplemented with *B. infantis*. The level of mesophilic and termophilic lactobacilli and lactococci was always higher than 6 logcfu/g, while enterobacteria, pseudomonads, yeasts and staphylococci were below the detection limit (data not shown).

**Figure 8:**
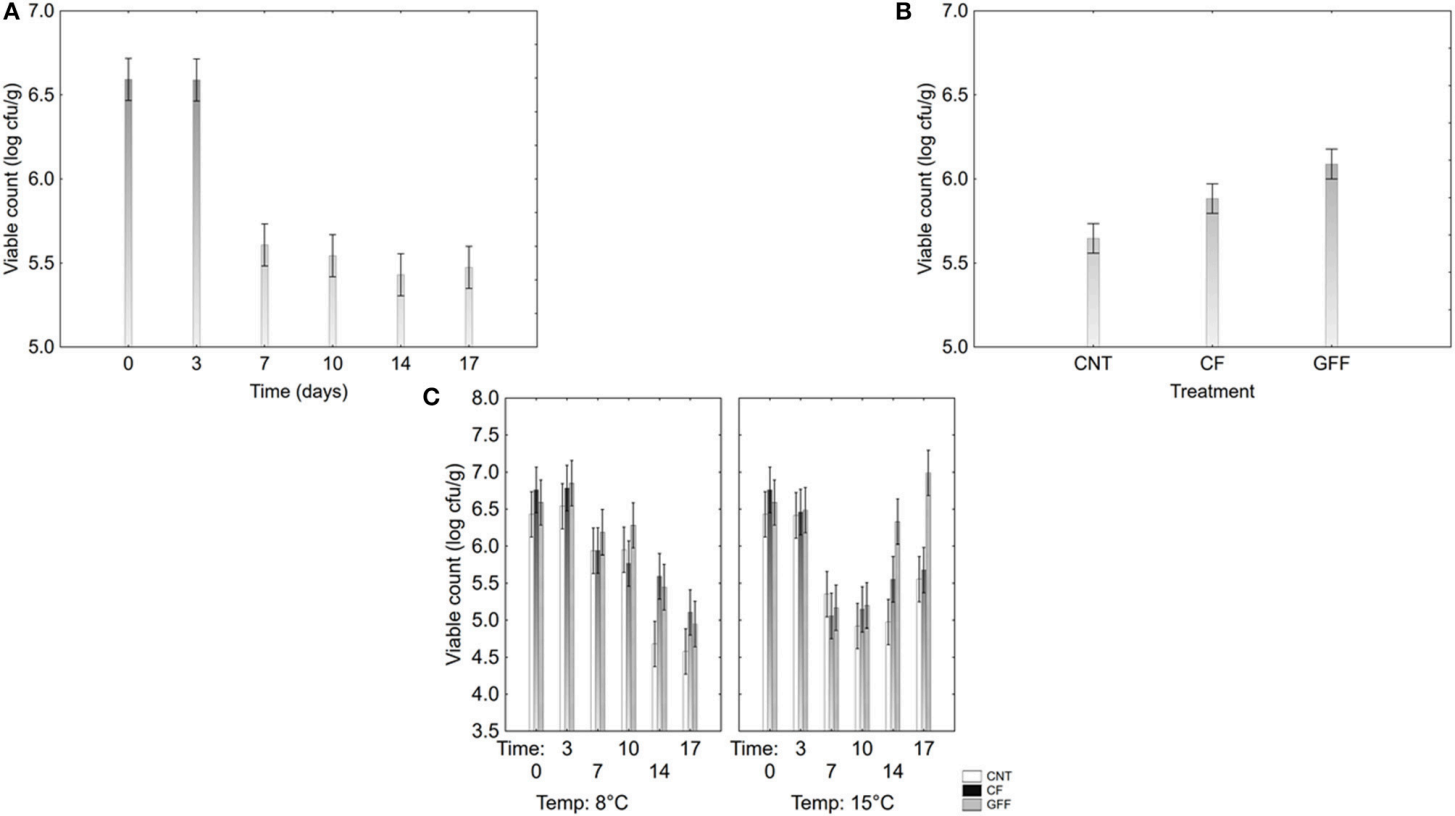
Decomposition of the statistical hypothesis of multifactorial ANOVA on the viable count of *B. infantis*. In the yogurt. **(A)**, effect of the storage time; **(B)**, effect of flour addition; **(C)**, interaction time^*^temperature^*^flour addition. CNT, without flour (control); CF, Control Flour; GF, Gluten Friendly Flour. The bars represent the 95%-confidence intervals.

The viable count of *B. infantis* was analyzed through a multifactorial ANOVA; the results are in Table 4 and in Figure 8. *B. infantis* was affected by time, temperature, and flour (Table 4).

**Table 4:** Standardized effects of treatment (addition of GF or CF), duration of storage (time) and storage temperature on the viable count of *B. infantis*.

	**SS**	**Degree of freedom**	**MS**	**Fishet-test**	***P*-level**
Intercept	2483.37	1	2483.37	54220.85	<0.0001
Time	18.78	5	3.76	82.01	<0.0001
Temperature	–^*^	–	–	–	–
Treatment	2.35	2	1.17	25.62	<0.0001
Time^*^Temp.	9.41	5	1.88	41.07	<0.0001
Time^*^Treatment	2.30	10	0.23	5.02	0.0001
Temp.^*^Treatment	–	–	–	–	–
Time^*^Temp.^*^Treat	1.59	10	0.16	3.48	0.003

*SS, sum of squares; MS, mean square residual*.

* *Not significant*.

A second output of multifactorial ANOVA is the decomposition of the statistical hypothesis, which shows the correlation of each factor or interactive term with the dependent variable. The decomposition of the statistical hypothesis for the viable count of *Bifidobacterium* has 3 outputs: correlation storage time vs. viable count (8A); qualitative correlation kind of flour vs. viable count (8B); actual data (8C). The decomposition of the statistical hypothesis, does not show effective or actual trends but it is a mathematical extrapolation of their statistical effect.

Figure 8A shows the effect of the storage time; as expected, time negatively acted on the viable count of *B. infantis*, as the cell number decreased within storage. Figure 8A shows the effect of the supplementation of flour; it does not report the viable count over time but it shows “mean” values for the entire running time. Flour supplementation exerted a positive effect, as in the sample with GF the analysis predicted the highest value and the lowest in the control; the sample supplemented with CF showed an intermediate trend (Figure 8B).

The decomposition of the statistical hypothesis for the interaction time/temperature/treatment shows when GF acted (Figure 8C). The positive effect of GF was found at 15°C (thermal abuse conditions), when it determined an increase of Bb02 in the last days of storage: the viable count of *B. infantis* was 7.0 log cfu/g in GF sample and 5.5–5.7 log cfu/g in the control and in CF sample.pH (3.8–4.0), Aw (0.98) and color did not undergo significant changes (data not shown).

## Discussion

The optimization of a product is a complex process, as many variables could play a significant role and affect the final output; in this paper, the optimization of synbiotic yogurt was done through a step-by-step approach as the proposed product (traditional yogurt supplemented with both *B. infantis* and GF flour) involves different microorganisms.

First, the effect of GF on the acidification kinetics was studied, as the main requirement of an ingredient is that it must not delay or significantly affect the performances of a starter culture (Speranza et al., 2018). The optimization was based on two parameters: ΔpH_max_, as a tool to measure the performances (acidification), and α, to highlight possible delays in the kinetic or a reversible inhibition.

The results show that the acidification kinetics of *L. bulgaricus* was significantly affected by the factors of the design (flour, inoculum, temperature). Namely, flour positively acted on acidification and this result was different from what found on *L. acidophilus*, which experienced a positive trend up to 2.5 g/l (Speranza et al., 2018). However, the most important result was the uncoupling between the performances of the microorganism in terms of pH decrease (ΔpH_max_) and the parameter α. The combinations resulting in the highest reduction of pH also caused a delay of the kinetic. To the best of our knowledge, this trend was never found on *L. bulgaricus* and *S. thermophilus* and represents a challenge, as it was not possible to find out a combination with the highest acidification and the lowest value of α. The lack of adequate acidification could lead to long fermentation times with unsuitable economic and hygienic consequences (Mohammadi et al., 2012).

Therefore, the optimization was done by using a risk-benefit approach. As a cut-off point, two criteria were set: acidification of 2–2.5, in order to attain a final pH of 4.0, and α of 6–7 h; as a result, the combination able to fulfill this requirement was as follows: flour 2.5 g/l; inoculum 6 log cfu/ml; temperature 37°C.

Inoculum size of probiotic bacteria is an important key factor to ensure sufficient viable cells in the final food product (Lourens-Hattingh and Viljoen, 2001). The controversial effect of the inoculum (increase of performances up to a break-point and then decrease of acidification) confirmed the preliminary reports on *L. acidophilus* La5 (Speranza et al., 2018) and was also reported by other authors; Khosravi-Darani et al. (2015) studied the effect of inoculum size in mono- and co-colture of *L. bulgaricus, L. acidophilus*, and *S. thermophilus* and found that *S. thermophilus* multiplication in yogurt was higher once a lower concentration of inoculum had been used. This effect was attributed to a possible competition for nutrients.

After the optimization, the effect of GF on the viability of the probiotic *Bifidobacterium* was assessed, as some preliminary reports (Bevilacqua et al., 2016; Costabile et al., 2017) highlighted the importance of GF (flours or bread) and pointed out a positive effect and a partial restoration of bifidobacteria in coeliac subjects, along with a protective effect and a lowering of death kinetic of *L. acidophilus* La-5 (Bevilacqua et al., 2016; Speranza et al., 2018).

The results of the second step confirmed the protective effect and the lowering of the death kinetic. However, the data of the last part, which surprisingly denoted a partial increase of bifidobacteria in the last days of the storage, highlighted that the effect of GF was not a mere protection, but also a possible stimulation of bifidobacterial population (Costabile et al., 2017).

GF technology is based on the use of microwaves for few seconds on hydrated wheat kernels; this process, coupled with rest times and water evaporation, probably induces a modification in secondary and tertiary structures of proteins. As a result of this change, the different spatial configuration of amino acids is able to drastically reduce the immunogenicity (Landriscina et al., 2017) and exerts a positive effect on some Gram-positive bacteria (namely lactobacilli and bifidobacteria).

Previously, it was suggested that the gluten modified by GF technology could be used as an alternative source under stressful conditions (Speranza et al., 2018), like those encountered by bifidobacteria after a prolonged storage at 15°C.

A key-factor for the optimization/design of functional foods is the viable count of probiotic. The traditional threshold is 10^6^ cfu/g or cfu/ml by the Italian legislation (Fortina, 2007) and recently increased to 10^7^ cfu/g or 10^9^ *per* day (Rosburg et al., 2010; Italian Ministry of Health, 2013). The level of *B. infantis* for both the second and the third assay is lower than this break-point; namely in the second step the inoculum was 7.7 log cfu/ml, and this assured a probiotic shelf-life for 1 day in the control and in the sample supplemented with CF and 2 days for the sample supplemented for GF. The low inoculum affected the duration of shelf life; however, the main goal of this step was not the evaluation of shelf life but a focus on the effect of GF on the viability.

In the validation in synbiotic yogurt, the inoculum was lower (6.5–6.8 log cfu/g) as preliminary reports suggested that in complex systems GF could promote or enhance growth; thus, a compromise between the viable count required for a viability test (7–8 log cfu/g) and a growth assay (4–5 log cfu/g) was chosen. Therefore, this step, as previously reported, was not aimed at defining the shelf life but at studying the effect of GF in a complex system. Further investigations are required to define shelf life by adding to the yogurt higher concentration of *B. infantis* (8–9 log cfu/g).

In addition, the results of 2nd and 3rd step both suggest that the supplementation of GFF at 2.5 g/l in a yogurt is a good compromise between technological performances of starter cultures and prolongation/enhancement of the viability of the probiotic microorganism *B. infantis*. However, the design of this synbiotic yogurt should be completed with the optimization of the inoculum of *B. infantis* after the fermentation to fulfill the basic requirements of law (probiotic at least 7 log cfu/g for the entire storage time).

This paper proposes a DoE approach to produce a traditional yogurt supplemented with GF and a probiotic *Bifidobacterium*. The most important findings can be summarized as follows:

- The flour is responsible on an uncoupling on the fermentation kinetics of the starter microorganisms of the yogurt (increase of acidification and induction of a delay in the kinetic). Thus, it is not advisable to use flour amounts > 2.5 g/l.
- The supplementation of GF exerted a positive effect in a simple system, as it prolonged the viability of *B. infantis*.
- It is possible produce a synbiotic yogurt, fermented with *L. delbrueckii* subsp. *bulgaricus* and *S. thermophilus* and supplemented with *B. infantis* and a prebiotic-like compound (GF). GF was able to cause an increase of bifidobacteria in the last days of storage.

In conclusion, this paper suggests a way to produce a synbiotic yogurt containing bifidobacterial and GF; the combination of these two factors could prolong and enhance the viability of the probiotic. The protocol hereby proposed also shows a way to counteract the drawback of bifidus pathway (production of acetic acid), by using the traditional starter cultures of yogurt. The combination of all these data suggests that the approach proposed in this paper is a promising way; however, further efforts are required to translate these preliminary data in an effective protocol to produce a new functional food. Some requirements have to be fulfilled: the definition of a unique death kinetic of the probiotic, along with a focus on the mode of action of GF, in order to evaluate and define a commercial shelf life as requested by the Regulatory Agencies.

## Author Contributions

AB, MS, CL, and MC conceived the study. AB, BS, DC, and MC designed the experiments. CL prepared the GF. BS, and DC performed the experiments. AB performed the statistic and wrote the paper. CL funded the research. All authors reviewed the paper.

### Conflict of Interest Statement

CL is the inventor of the following patents “Method for the detoxification of gluten proteins from grains of cereals. Patent Cooperation Treaty PCT/IB2013/000797” and “Methods for the detoxification of gluten proteins from grains of cereals and related medical uses. Italian priority patent n° 102015000084813 filed on 17.12.15.” The remaining authors declare that the research was conducted in the absence of any commercial or financial relationships that could be construed as a potential conflict of interest.
